# 14-Eth­oxy-4,6-dimethyl-9-phenyl-8,12-dioxa-4,6-diaza­tetra­cyclo­[8.8.0.0^2,7^.0^13,18^]octa­deca-2(7),13,15,17-tetra­ene-3,5,11-trione

**DOI:** 10.1107/S160053681205009X

**Published:** 2012-12-12

**Authors:** G. Jagadeesan, D. Kannan, M. Bakthadoss, S. Aravindhan

**Affiliations:** a Department of Physics, Presidency College, Chennai 600 005, India; b Department of Organic Chemistry, University of Madras, Guindy Campus, Chennai 600 025, India

## Abstract

In the title compound, C_23_H_20_N_2_O_6_, the fused pyrone and pyran rings each adopt a sofa conformation. The dihedral angle between the mean planes of the pyran and phenyl rings is 61.9 (1)°. In the crystal, mol­ecules are linked by two pairs of C—H⋯O hydrogen bonds, forming dimers. These dimers are linked *via* a third C—H⋯O hydrogen bond, forming a two-dimensional network parallel to (10-2).

## Related literature

For the biological activity of pyran­ocoumarin compounds, see: Kawaii *et al.* (2001 ▶); Hossain *et al.* (1996 ▶); Goel *et al.* (1997 ▶); Su *et al.* (2009 ▶); Xu *et al.* (2006 ▶). For anti-filarial activity studies, see: Casley-Smith *et al.* (1993 ▶). For their enzyme inhibitory activity, see: Pavao *et al.* (2002 ▶). For asymmetry parameters, see: Nardelli (1983 ▶).
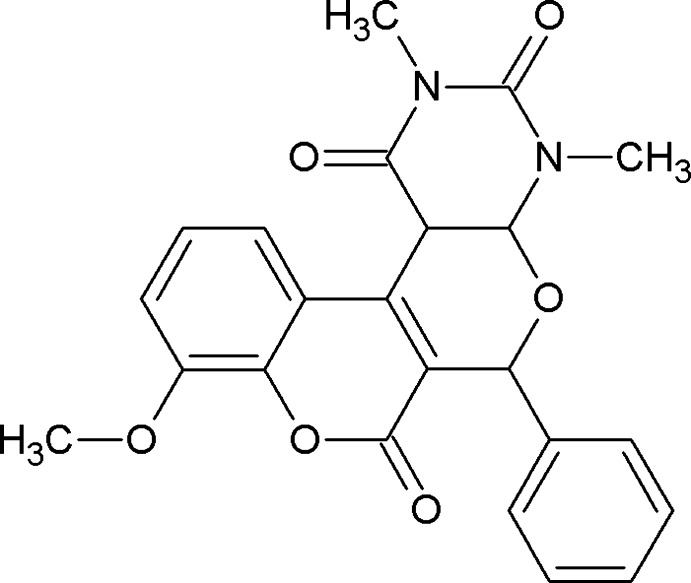


## Experimental

### 

#### Crystal data

C_23_H_20_N_2_O_6_
*M* *_r_* = 420.41
Monoclinic, 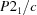

*a* = 16.8362 (9) Å
*b* = 8.1692 (4) Å
*c* = 14.4400 (8) Å
β = 98.000 (3)°
*V* = 1966.72 (18) Å^3^
*Z* = 4
Mo *K*α radiation
μ = 0.10 mm^−1^
*T* = 293 K
0.25 × 0.20 × 0.20 mm

#### Data collection

Bruker Kappa APEXII CCD diffractometer
Absorption correction: multi-scan (*SADABS*; Bruker 2004 ▶) *T*_min_ = 0.979, *T*_max_ = 0.983
19858 measured reflections
4247 independent reflections
2943 reflections with *I* > 2σ(*I*)
*R*_int_ = 0.039

#### Refinement

*R*[*F* ^2^ > 2σ(*F* ^2^)] = 0.046
*wR*(*F* ^2^) = 0.130
*S* = 1.04
4247 reflections
281 parameters
H-atom parameters constrained
Δρ_max_ = 0.28 e Å^−3^
Δρ_min_ = −0.19 e Å^−3^

### 

Data collection: *APEX2* (Bruker, 2004 ▶); cell refinement: *APEX2* and *SAINT* (Bruker, 2004 ▶); data reduction: *SAINT* and *XPREP* (Bruker, 2004 ▶); program(s) used to solve structure: *SHELXS97* (Sheldrick, 2008 ▶); program(s) used to refine structure: *SHELXL97* (Sheldrick, 2008 ▶); molecular graphics: *ORTEP-3 for Windows* (Farrugia, 2012 ▶); software used to prepare material for publication: *PLATON* (Spek, 2009 ▶).

## Supplementary Material

Crystal structure: contains datablock(s) I, global. DOI: 10.1107/S160053681205009X/su2537sup1.cif

Structure factors: contains datablock(s) I. DOI: 10.1107/S160053681205009X/su2537Isup2.hkl

Supplementary material file. DOI: 10.1107/S160053681205009X/su2537Isup3.cml

Additional supplementary materials:  crystallographic information; 3D view; checkCIF report

## Figures and Tables

**Table 1:** Hydrogen-bond geometry (Å, °)

*D*—H⋯*A*	*D*—H	H⋯*A*	*D*⋯*A*	*D*—H⋯*A*
C4—H4⋯O1^i^	0.93	2.59	3.514 (3)	172
C5—H5⋯O3^i^	0.93	2.45	3.361 (3)	165
C18—H18⋯O6^ii^	0.93	2.56	3.215 (3)	128

Symmetry codes: (i) 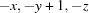; (ii) 
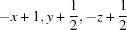
.

**Fig. 1.**
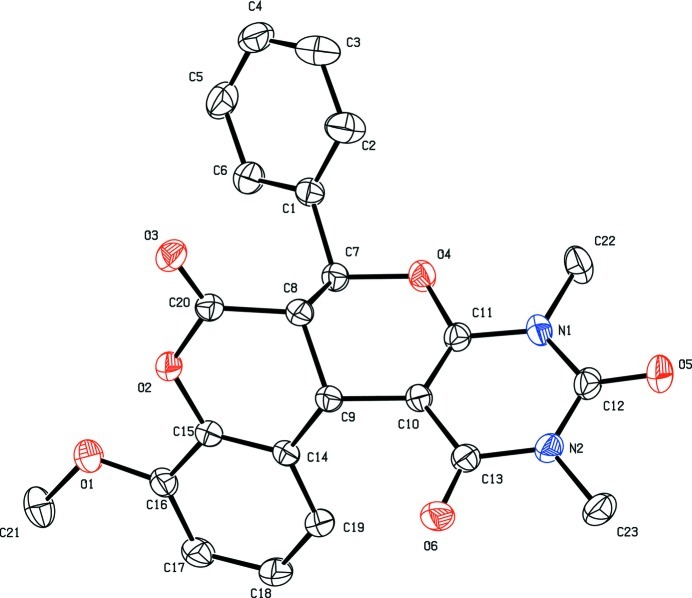
The molecular structure of the title molecule, with atom lables. Displacement ellipsoids are drawn at the 30% probability level (H atoms have been omitted for clarity).

**Fig. 2.**
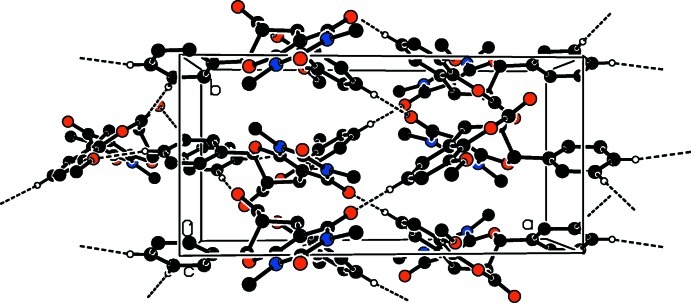
A view along the c axis of the crystal packing of the title compound. The C-H···O hydrogen bonds are shown as dashed lines. For the sake of clarity, H atoms not involved in these interactions have been omitted.

## supplementary crystallographic information

### Comment

Coumarin derivatives show strong activity against cancer cell lines (Kawaii *et al.*, 2001) and exhibit monoamine oxidase inhibitory activity (Hossain *et al.*, 1996). Antiulcer activity of some naturally occurring pyranocoumarins has been reported (Goel *et al.*, 1997). They also show anti-hepatitis B virus, anti-filarial (Casley-Smith *et al.*, 1993) and cytotoxic activities (Su *et al.*, 2009) and anti-TB activity (Xu *et al.*, 2006). One natural source coumarin derivative, Chalepin, inhibits the glyceraldehyde-3-phosphate dehydrogenase of parasites (Protein Data Bank ID code 1 K3T) (Pavao *et al.*, 2002). Herein, we report on the crystal structure of the title coumarin derivative.

In the title molecule (Fig. 1) the six-membered pyrone ring of the coumarin ring system [DS (C9) = 0.163 (1) Å and D2 (C9—C8) = 0.029 (1) Å] and the pyran ring [DS (C8) = 0.065 (1) Å and D2 (C8—C7) = 0.075 (1) Å] both adopt a sofa conformation defined by the above asymmetry parameters (Nardelli, 1983). The mean plane of the pyran ring and the phenyl ring are tilted with respect to one another with a dihedral angle of 61.9 (1) °. The torsion angles H9—C9—C8—H8 = 51 (2)° and H8—C8—C7—H7 = 175.12 (2)°, define the ring fusions involving the in the fused pyrone and pyran ring system of the coumarin moiety.

In the crystal, molecules are linked by two pairs of C-H···O hydrogen bonds to form dimers. These dimers are linked via a third C-H···O hydrogen bond forming a two-dimensional network parallel to (10-2) [Table 1 and Fig. 2].

### Experimental

A mixture of 2-ethoxy-6-formylphenyl (2E)-3-phenylprop-2-enoate (0.296 g, 1 mmol) and *N*,*N*-dimethylbarbituric acid (0.156 g, 1 mmol) was placed in a round bottom flask and melted at 453 K for 1 h. After completion of the reaction, as indicated by TLC, the crude product was washed with 5 ml of an ethylacetate and hexane mixture (1:49 ratio) which successfully provided the pure product in 92% yield as a colourless solid. Diffraction quality crystals were obtained by slow evaporation of a solution in ethyl acetate.

### Refinement

All the H atoms were positioned geometrically and constrained to ride on their parent atom: C–H = 0.93, 0.98 and 0.96 Å for aromatic, methine and methyl H atoms, respectively, with U_iso_(H) = 1.5U_eq_(C) for methyl H atoms and = 1.2U_eq_(C) for other H atoms.

### Crystal data

**Table:**

C_23_H_20_N_2_O_6_	*F*(000) = 880
*M**_r_* = 420.41	*D*_x_ = 1.420 Mg m^−^^3^
Monoclinic, *P*2_1_/*c*	Mo *K*α radiation, λ = 0.71073 Å
Hall symbol: -P 2ybc	Cell parameters from 8834 reflections
*a* = 16.8362 (9) Å	θ = 2.1–31.2°
*b* = 8.1692 (4) Å	µ = 0.10 mm^−^^1^
*c* = 14.4400 (8) Å	*T* = 293 K
β = 98.000 (3)°	Block, colourless
*V* = 1966.72 (18) Å^3^	0.25 × 0.20 × 0.20 mm
*Z* = 4	

### Data collection

**Table:**

Bruker Kappa APEXII CCD diffractometer	4247 independent reflections
Radiation source: fine-focus sealed tube	2943 reflections with *I* > 2σ(*I*)
Graphite monochromator	*R*_int_ = 0.039
ω and φ scan	θ_max_ = 26.9°, θ_min_ = 1.2°
Absorption correction: multi-scan (*SADABS*; Bruker 2004)	*h* = −21→21
*T*_min_ = 0.979, *T*_max_ = 0.983	*k* = −10→10
19858 measured reflections	*l* = −18→16

### Refinement

**Table:**

Refinement on *F*^2^	Secondary atom site location: difference Fourier map
Least-squares matrix: full	Hydrogen site location: inferred from neighbouring sites
*R*[*F*^2^ > 2σ(*F*^2^)] = 0.046	H-atom parameters constrained
*wR*(*F*^2^) = 0.130	*w* = 1/[σ^2^(*F*_o_^2^) + (0.0545*P*)^2^ + 0.6922*P*] where *P* = (*F*_o_^2^ + 2*F*_c_^2^)/3
*S* = 1.04	(Δ/σ)_max_ < 0.001
4247 reflections	Δρ_max_ = 0.28 e Å^−^^3^
281 parameters	Δρ_min_ = −0.19 e Å^−^^3^
0 restraints	Extinction correction: *SHELXL97* (Sheldrick, 2008), Fc^*^=kFc[1+0.001xFc^2^λ^3^/sin(2θ)]^-1/4^
Primary atom site location: structure-invariant direct methods	Extinction coefficient: 0.0027 (7)

### Special details

**Table:**

Geometry. All e.s.d.'s (except the e.s.d. in the dihedral angle between two l.s. planes) are estimated using the full covariance matrix. The cell e.s.d.'s are taken into account individually in the estimation of e.s.d.'s in distances, angles and torsion angles; correlations between e.s.d.'s in cell parameters are only used when they are defined by crystal symmetry. An approximate (isotropic) treatment of cell e.s.d.'s is used for estimating e.s.d.'s involving l.s. planes.
Refinement. Refinement of *F*^2^ against ALL reflections. The weighted *R*-factor *wR* and goodness of fit *S* are based on *F*^2^, conventional *R*-factors *R* are based on *F*, with *F* set to zero for negative *F*^2^. The threshold expression of *F*^2^ > σ(*F*^2^) is used only for calculating *R*-factors(gt) *etc*. and is not relevant to the choice of reflections for refinement. *R*-factors based on *F*^2^ are statistically about twice as large as those based on *F*, and *R*- factors based on ALL data will be even larger.

### Fractional atomic coordinates and isotropic or equivalent isotropic displacement parameters (Å^2^)

**Table:**

	*x*	*y*	*z*	*U*_iso_*/*U*_eq_	
O4	0.16385 (8)	0.54434 (17)	0.34721 (9)	0.0456 (4)	
O2	0.22837 (7)	0.36035 (17)	0.07300 (9)	0.0438 (3)	
O3	0.12638 (8)	0.20829 (18)	0.09730 (10)	0.0513 (4)	
O6	0.41821 (8)	0.2943 (2)	0.41399 (10)	0.0548 (4)	
O5	0.29499 (10)	0.5286 (2)	0.64093 (10)	0.0632 (4)	
N1	0.23397 (10)	0.5455 (2)	0.49074 (11)	0.0433 (4)	
O1	0.29820 (9)	0.5214 (2)	−0.04780 (10)	0.0547 (4)	
N2	0.35919 (9)	0.4205 (2)	0.52645 (11)	0.0450 (4)	
C13	0.36037 (11)	0.3696 (2)	0.43419 (13)	0.0412 (4)	
C11	0.23195 (11)	0.4981 (2)	0.39956 (13)	0.0379 (4)	
C14	0.32631 (10)	0.4522 (2)	0.20279 (13)	0.0369 (4)	
C10	0.29172 (10)	0.4137 (2)	0.36840 (13)	0.0367 (4)	
C16	0.33429 (11)	0.5327 (2)	0.04229 (14)	0.0427 (5)	
C20	0.17880 (10)	0.2965 (2)	0.13077 (13)	0.0387 (4)	
C15	0.29621 (10)	0.4502 (2)	0.10910 (13)	0.0378 (4)	
C9	0.28330 (10)	0.3522 (2)	0.26942 (12)	0.0365 (4)	
H9	0.3045	0.2404	0.2707	0.044*	
C8	0.19371 (10)	0.3446 (2)	0.23246 (13)	0.0378 (4)	
H8	0.1691	0.2618	0.2685	0.045*	
C7	0.15484 (11)	0.5103 (2)	0.24763 (13)	0.0386 (4)	
H7	0.1821	0.5959	0.2164	0.046*	
C1	0.06624 (11)	0.5170 (2)	0.21317 (14)	0.0401 (4)	
C12	0.29624 (12)	0.4995 (3)	0.55866 (14)	0.0460 (5)	
C22	0.16917 (14)	0.6433 (3)	0.52058 (15)	0.0566 (6)	
H22A	0.1808	0.6647	0.5864	0.085*	
H22C	0.1196	0.5842	0.5079	0.085*	
H22B	0.1647	0.7450	0.4870	0.085*	
C17	0.40429 (12)	0.6166 (3)	0.07246 (15)	0.0515 (5)	
H17	0.4313	0.6705	0.0294	0.062*	
C19	0.39570 (11)	0.5407 (3)	0.23118 (15)	0.0478 (5)	
H19	0.4163	0.5459	0.2943	0.057*	
C18	0.43425 (12)	0.6208 (3)	0.16645 (16)	0.0555 (6)	
H18	0.4811	0.6785	0.1863	0.067*	
C6	0.04019 (13)	0.5835 (3)	0.12795 (15)	0.0547 (6)	
H6	0.0773	0.6274	0.0928	0.066*	
C21	0.33837 (15)	0.5946 (3)	−0.11801 (15)	0.0631 (6)	
H21A	0.3071	0.5791	−0.1782	0.095*	
H21B	0.3900	0.5445	−0.1174	0.095*	
H21C	0.3451	0.7096	−0.1056	0.095*	
C23	0.42923 (13)	0.3828 (3)	0.59566 (16)	0.0614 (6)	
H23A	0.4690	0.3278	0.5654	0.092*	
H23B	0.4132	0.3133	0.6434	0.092*	
H23C	0.4513	0.4825	0.6234	0.092*	
C2	0.01032 (13)	0.4541 (3)	0.26413 (19)	0.0633 (7)	
H2	0.0267	0.4096	0.3230	0.076*	
C4	−0.09540 (13)	0.5224 (3)	0.14226 (19)	0.0647 (7)	
H4	−0.1496	0.5229	0.1181	0.078*	
C5	−0.04080 (15)	0.5868 (3)	0.09263 (18)	0.0679 (7)	
H5	−0.0575	0.6337	0.0345	0.082*	
C3	−0.07031 (14)	0.4565 (3)	0.2283 (2)	0.0738 (8)	
H3	−0.1077	0.4129	0.2631	0.089*	

### Atomic displacement parameters (Å^2^)

**Table:**

	*U*^11^	*U*^22^	*U*^33^	*U*^12^	*U*^13^	*U*^23^
O4	0.0445 (7)	0.0561 (8)	0.0357 (7)	0.0139 (6)	0.0034 (6)	−0.0044 (6)
O2	0.0415 (7)	0.0573 (8)	0.0326 (7)	−0.0085 (6)	0.0054 (6)	−0.0022 (6)
O3	0.0488 (8)	0.0552 (8)	0.0481 (9)	−0.0117 (7)	0.0006 (7)	−0.0065 (7)
O6	0.0386 (7)	0.0730 (10)	0.0522 (9)	0.0106 (7)	0.0048 (6)	0.0051 (8)
O5	0.0691 (10)	0.0884 (12)	0.0320 (8)	−0.0072 (9)	0.0067 (7)	−0.0053 (8)
N1	0.0472 (9)	0.0523 (10)	0.0315 (9)	0.0016 (7)	0.0097 (7)	−0.0019 (7)
O1	0.0533 (8)	0.0768 (10)	0.0347 (8)	−0.0044 (7)	0.0084 (6)	0.0080 (7)
N2	0.0401 (9)	0.0587 (10)	0.0347 (9)	−0.0063 (8)	0.0003 (7)	0.0046 (8)
C13	0.0382 (10)	0.0483 (11)	0.0371 (11)	−0.0051 (9)	0.0049 (8)	0.0061 (9)
C11	0.0390 (9)	0.0414 (10)	0.0330 (10)	−0.0023 (8)	0.0034 (8)	0.0026 (8)
C14	0.0316 (9)	0.0449 (10)	0.0352 (10)	0.0033 (8)	0.0079 (7)	−0.0010 (8)
C10	0.0362 (9)	0.0423 (10)	0.0318 (10)	−0.0009 (8)	0.0056 (7)	0.0034 (8)
C16	0.0411 (10)	0.0526 (11)	0.0354 (11)	0.0044 (9)	0.0088 (8)	0.0028 (9)
C20	0.0347 (9)	0.0411 (10)	0.0400 (11)	0.0022 (8)	0.0042 (8)	−0.0019 (8)
C15	0.0325 (9)	0.0444 (10)	0.0371 (11)	0.0014 (8)	0.0071 (8)	−0.0011 (8)
C9	0.0326 (9)	0.0421 (10)	0.0350 (10)	0.0026 (7)	0.0054 (7)	0.0005 (8)
C8	0.0351 (9)	0.0427 (10)	0.0363 (10)	−0.0020 (8)	0.0068 (7)	0.0015 (8)
C7	0.0383 (9)	0.0443 (10)	0.0331 (10)	0.0024 (8)	0.0050 (8)	0.0021 (8)
C1	0.0372 (10)	0.0419 (10)	0.0414 (11)	0.0068 (8)	0.0059 (8)	−0.0018 (8)
C12	0.0503 (11)	0.0537 (12)	0.0335 (11)	−0.0109 (9)	0.0044 (9)	0.0027 (9)
C22	0.0682 (14)	0.0626 (14)	0.0412 (12)	0.0153 (11)	0.0158 (10)	−0.0050 (10)
C17	0.0449 (11)	0.0635 (14)	0.0485 (13)	−0.0078 (10)	0.0150 (9)	0.0090 (10)
C19	0.0376 (10)	0.0648 (13)	0.0407 (11)	−0.0063 (9)	0.0049 (8)	−0.0020 (10)
C18	0.0406 (11)	0.0730 (15)	0.0534 (14)	−0.0165 (10)	0.0087 (9)	−0.0006 (11)
C6	0.0477 (11)	0.0738 (15)	0.0412 (12)	0.0026 (11)	0.0020 (9)	0.0061 (11)
C21	0.0835 (17)	0.0693 (15)	0.0397 (13)	−0.0016 (13)	0.0199 (12)	0.0094 (11)
C23	0.0477 (12)	0.0874 (17)	0.0450 (13)	−0.0090 (12)	−0.0078 (10)	0.0099 (12)
C2	0.0467 (12)	0.0761 (16)	0.0682 (16)	0.0043 (11)	0.0122 (11)	0.0215 (13)
C4	0.0387 (11)	0.0757 (16)	0.0765 (18)	0.0069 (11)	−0.0033 (12)	−0.0295 (14)
C5	0.0590 (14)	0.0907 (19)	0.0489 (14)	0.0113 (13)	−0.0111 (11)	−0.0079 (13)
C3	0.0445 (12)	0.0807 (18)	0.099 (2)	−0.0016 (12)	0.0204 (14)	0.0022 (16)

### Geometric parameters (Å, º)

**Table:**

O4—C11	1.337 (2)	C7—C1	1.506 (2)
O4—C7	1.452 (2)	C7—H7	0.9800
O2—C20	1.363 (2)	C1—C6	1.361 (3)
O2—C15	1.396 (2)	C1—C2	1.373 (3)
O3—C20	1.189 (2)	C22—H22A	0.9600
O6—C13	1.221 (2)	C22—H22C	0.9600
O5—C12	1.215 (2)	C22—H22B	0.9600
N1—C11	1.368 (2)	C17—C18	1.381 (3)
N1—C12	1.384 (3)	C17—H17	0.9300
N1—C22	1.465 (3)	C19—C18	1.376 (3)
O1—C16	1.360 (2)	C19—H19	0.9300
O1—C21	1.426 (2)	C18—H18	0.9300
N2—C12	1.376 (3)	C6—C5	1.388 (3)
N2—C13	1.398 (3)	C6—H6	0.9300
N2—C23	1.468 (2)	C21—H21A	0.9600
C13—C10	1.436 (2)	C21—H21B	0.9600
C11—C10	1.348 (3)	C21—H21C	0.9600
C14—C15	1.377 (3)	C23—H23A	0.9600
C14—C19	1.386 (3)	C23—H23B	0.9600
C14—C9	1.520 (2)	C23—H23C	0.9600
C10—C9	1.503 (3)	C2—C3	1.384 (3)
C16—C17	1.381 (3)	C2—H2	0.9300
C16—C15	1.403 (3)	C4—C5	1.349 (4)
C20—C8	1.507 (3)	C4—C3	1.366 (4)
C9—C8	1.530 (2)	C4—H4	0.9300
C9—H9	0.9800	C5—H5	0.9300
C8—C7	1.533 (3)	C3—H3	0.9300
C8—H8	0.9800		
			
C11—O4—C7	117.97 (14)	C6—C1—C2	118.38 (19)
C20—O2—C15	120.75 (14)	C6—C1—C7	119.54 (18)
C11—N1—C12	121.34 (17)	C2—C1—C7	122.06 (18)
C11—N1—C22	121.18 (16)	O5—C12—N2	122.78 (19)
C12—N1—C22	117.46 (17)	O5—C12—N1	121.7 (2)
C16—O1—C21	117.29 (17)	N2—C12—N1	115.52 (17)
C12—N2—C13	125.11 (16)	N1—C22—H22A	109.5
C12—N2—C23	116.86 (17)	N1—C22—H22C	109.5
C13—N2—C23	118.00 (17)	H22A—C22—H22C	109.5
O6—C13—N2	119.66 (17)	N1—C22—H22B	109.5
O6—C13—C10	124.30 (18)	H22A—C22—H22B	109.5
N2—C13—C10	116.04 (17)	H22C—C22—H22B	109.5
O4—C11—C10	125.25 (17)	C18—C17—C16	120.08 (19)
O4—C11—N1	111.64 (16)	C18—C17—H17	120.0
C10—C11—N1	123.11 (17)	C16—C17—H17	120.0
C15—C14—C19	118.45 (18)	C18—C19—C14	120.33 (19)
C15—C14—C9	118.22 (16)	C18—C19—H19	119.8
C19—C14—C9	123.30 (17)	C14—C19—H19	119.8
C11—C10—C13	118.49 (17)	C19—C18—C17	120.93 (19)
C11—C10—C9	120.83 (16)	C19—C18—H18	119.5
C13—C10—C9	120.39 (16)	C17—C18—H18	119.5
O1—C16—C17	125.70 (18)	C1—C6—C5	121.1 (2)
O1—C16—C15	116.05 (17)	C1—C6—H6	119.4
C17—C16—C15	118.25 (18)	C5—C6—H6	119.4
O3—C20—O2	117.79 (17)	O1—C21—H21A	109.5
O3—C20—C8	124.59 (18)	O1—C21—H21B	109.5
O2—C20—C8	117.62 (15)	H21A—C21—H21B	109.5
C14—C15—O2	122.90 (16)	O1—C21—H21C	109.5
C14—C15—C16	121.94 (17)	H21A—C21—H21C	109.5
O2—C15—C16	115.09 (16)	H21B—C21—H21C	109.5
C10—C9—C14	115.59 (15)	N2—C23—H23A	109.5
C10—C9—C8	107.66 (14)	N2—C23—H23B	109.5
C14—C9—C8	109.55 (14)	H23A—C23—H23B	109.5
C10—C9—H9	107.9	N2—C23—H23C	109.5
C14—C9—H9	107.9	H23A—C23—H23C	109.5
C8—C9—H9	107.9	H23B—C23—H23C	109.5
C20—C8—C9	111.91 (15)	C1—C2—C3	120.3 (2)
C20—C8—C7	110.65 (15)	C1—C2—H2	119.8
C9—C8—C7	109.51 (15)	C3—C2—H2	119.8
C20—C8—H8	108.2	C5—C4—C3	119.3 (2)
C9—C8—H8	108.2	C5—C4—H4	120.3
C7—C8—H8	108.2	C3—C4—H4	120.3
O4—C7—C1	106.40 (15)	C4—C5—C6	120.3 (2)
O4—C7—C8	108.85 (14)	C4—C5—H5	119.9
C1—C7—C8	114.07 (15)	C6—C5—H5	119.9
O4—C7—H7	109.1	C4—C3—C2	120.6 (2)
C1—C7—H7	109.1	C4—C3—H3	119.7
C8—C7—H7	109.1	C2—C3—H3	119.7

### Hydrogen-bond geometry (Å, º)

**Table:**

*D*—H···*A*	*D*—H	H···*A*	*D*···*A*	*D*—H···*A*
C4—H4···O1^i^	0.93	2.59	3.514 (3)	172
C5—H5···O3^i^	0.93	2.45	3.361 (3)	165
C18—H18···O6^ii^	0.93	2.56	3.215 (3)	128

Symmetry codes: (i) −*x*, −*y*+1, −*z*; (ii) −*x*+1, *y*+1/2, −*z*+1/2.
